# GapMap: Enabling Comprehensive Autism Resource Epidemiology

**DOI:** 10.2196/publichealth.7150

**Published:** 2017-05-04

**Authors:** Nikhila Albert, Jena Daniels, Jessey Schwartz, Michael Du, Dennis P Wall

**Affiliations:** ^1^ Department of Pediatrics Division of Systems Medicine Stanford University Stanford, CA United States; ^2^ Department of Computer Science Princeton University Princeton, NJ United States; ^3^ Department of Biomedical Data Science Stanford University Stanford, CA United States

**Keywords:** autism, autism spectrum disorder, crowdsourcing, prevalence, resources, epidemiology

## Abstract

**Background:**

For individuals with autism spectrum disorder (ASD), finding resources can be a lengthy and difficult process. The difficulty in obtaining global, fine-grained autism epidemiological data hinders researchers from quickly and efficiently studying large-scale correlations among ASD, environmental factors, and geographical and cultural factors.

**Objective:**

The objective of this study was to define resource load and resource availability for families affected by autism and subsequently create a platform to enable a more accurate representation of prevalence rates and resource epidemiology.

**Methods:**

We created a mobile application, GapMap, to collect locational, diagnostic, and resource use information from individuals with autism to compute accurate prevalence rates and better understand autism resource epidemiology. GapMap is hosted on AWS S3, running on a React and Redux front-end framework. The backend framework is comprised of an AWS API Gateway and Lambda Function setup, with secure and scalable end points for retrieving prevalence and resource data, and for submitting participant data. Measures of autism resource scarcity, including resource load, resource availability, and resource gaps were defined and preliminarily computed using simulated or scraped data.

**Results:**

The average distance from an individual in the United States to the nearest diagnostic center is approximately 182 km (50 miles), with a standard deviation of 235 km (146 miles). The average distance from an individual with ASD to the nearest diagnostic center, however, is only 32 km (20 miles), suggesting that individuals who live closer to diagnostic services are more likely to be diagnosed.

**Conclusions:**

This study confirmed that individuals closer to diagnostic services are more likely to be diagnosed and proposes GapMap, a means to measure and enable the alleviation of increasingly overburdened diagnostic centers and resource-poor areas where parents are unable to diagnose their children as quickly and easily as needed. GapMap will collect information that will provide more accurate data for computing resource loads and availability, uncovering the impact of resource epidemiology on age and likelihood of diagnosis, and gathering localized autism prevalence rates.

## Introduction

### Background

Autism spectrum disorder (ASD) has been attracting more interest recently, as a result of skyrocketing prevalence rates. Centers for Disease Control and Prevention (CDC) estimates in 2000 suggested that the condition affected 1 in 150 children; 10 years later, the rate rose to 1 in 68 children [1]. However, many present epidemiological studies suffer from small sample sizes and regional focuses [2-5]. For example, the most recent CDC prevalence estimates were based on a limited number of communities in only 11 states [6]. However, studies that estimate prevalence on broader geographic scales often rely on aggregate data that omit valuable regional prevalence rates. Although families report concerns as early as 1 year of age and reliable diagnoses can be made during early childhood [7], the average age of diagnosis for ASD in the United States is more than 4 years and an estimated quarter of children remain undiagnosed at 8 years of age [8-9]. Despite the considerable number of undiagnosed individuals who meet the criteria for autism [10], studies that calculate prevalence rates often do not include undiagnosed individuals [11]. This means that individuals without access to diagnostic centers for socioeconomic or geographic reasons are not reported, resulting in underrepresented statistics [12,13]. Instead, an ideal dataset would span a large geographic region (such as the United States) and maintain high specificity (to the city level).

Comprehensive regional autism prevalence rates would be extremely helpful for determining the true prevalence of autism and correlating genetic and environmental factors with higher standards of significance. In particular, comparing geographic trends in prevalence rates with autism resource epidemiology would be invaluable in revealing patient care deficits.

Whereas much effort has been focused on measuring autism incidence and prevalence rates, few have explored resource epidemiology. Yet, studies reveal alarming indicators of significant resource shortages. In metropolitan areas, parents may wait up to 18 months after initial suspicions for a final diagnosis [14]. For families of minority populations or lower socioeconomic status, this process may take even longer [15]. Children with autism in rural or resource-poor areas are diagnosed much later than their peers in resource-rich areas (if they are diagnosed at all) and face difficulties finding appropriate post diagnosis services [12].

Finding these resource gaps, regions in which there are limited diagnostic or treatment resources with respect to the demand, can support pushes for congressional change with hard data, allocate resources more efficiently, and provide information to emerging organizations and businesses to let them know where their services are most needed. These efforts can help reduce age at the first diagnosis and ensure that speech and behavioral therapies are started during critical periods when they are maximally impactful [16,17].

### Aims of This Study

The specific aims of this study were to (1) obtain an early approximation of the disconnect between autism resources and diagnosed individuals by determining the average distance between an individual with autism and the nearest diagnostic center based on public census data in the United States and the United Kingdom, (2) define useful metrics that can be used to determine whether a center is overloaded or whether a region is underserved, and (3) propose an online prototype to collect information pertaining to geographic variations of autism prevalence and the geographic resource utilization of autism resources.

Our proposed app, GapMap [18], will contribute to the growing field of participatory surveillance and epidemiology [19-21] by prospectively collecting locational, diagnostic, and resource use information from individuals with autism in order to compute accurate prevalence rates and better understand autism prevalence rates and resource epidemiology.

## Methods

### Computing the Average Distance From an Individual to the Nearest Diagnostic Center

A simulation of the average distance from an individual in the United States to the nearest diagnostic center was constructed using county-level 2015 Census Data [22] and a list of 840 developmental medical centers that were collected through Autism Speaks [23] and Autism Source [24]. County populations and land area were used to model randomly uniform distributions of individuals within county borders; individuals were then assigned to the nearest diagnostic center. The average and standard deviation were then computed for the distance from an individual to the nearest diagnostic center.

### Computing the Average Distance From an Individual With ASD to the Nearest Diagnostic Center

A simulation of the average distance from an individual with an ASD to the nearest diagnostic center was constructed for the United States and the United Kingdom. Data from the United Kingdom are comprehensively collected and publicly available, which allowed us to conduct a proof-of-concept study with a more complete dataset to highlight disparities between families with a clinical diagnosis of autism and autism-related resources. City-level geographic approximations of individuals with autism were obtained by screen scraping information from the Internet and social networks. Lists of 840 and 135 diagnostic centers in the United States and United Kingdom, respectively, were collected through Autism Speaks [23], Autism Source [24], and the National Autistic Society (in the United Kingdom) [25]. Individuals were similarly assigned to the nearest diagnostic center, and statistics were computed. Maps of the individuals and centers were generated for both countries.

### Computing Resource Load and Availability

Resource load was computed for 840 diagnostic centers in the United States using county-level 2015 Census Data [22-24]. Individuals were randomly assigned to a center near them, where nearby centers were defined as all the centers within a radius of 25, 100, 500, or 3000 km (the smallest radius was chosen so that at least one center existed within that distance). The maximum and average distance an individual traveled to each center was calculated in addition to the resource load.

Resource load was calculated based on an approximation of the number of caseloads per clinician in a year and an estimation of the number of clinicians at a single diagnostic center. These numbers vary greatly by clinician, services, and the size of the diagnostic centers, but families are most often referred to general practitioners, pediatricians, speech and language therapists, psychologists, and psychiatrists [26]. We calculated our statistics based on an estimate that the average diagnostic center is composed of 5 specialists who can attend to about 200 patients each per year. The actual number is likely lower. Two hospitals in Ohio, both of which have 4-5 clinicians on their teams for diagnostics, experience months-long waitlists, with an estimated 40-100 referrals for ASD each month and about 698-1100 patients with ASD who need ongoing care per year [27]. A school district in Texas, staffs 52 diagnosticians and 45 speech language pathologists across about 60 schools, and estimates that each diagnostician has a caseload of 87.5 students per year and each speech language pathologist has a caseload of 53.3 students per year [28], yet the American Speech-Language-Hearing Association estimates that the median monthly caseload of a speech language pathologist working full-time in the United States was 47 cases per month [29]. For analysis, we set the percent of individuals in need of an autism screening set to 0.195% (given that 6.5% of individuals are at an age appropriate for diagnosis from US Census Data, with 3% of those children needing an autism screening).

Resource availability statistics were also computed (Equation 2 in “Results” section) for each county in the United States and used to generate a heatmap. The results from the previous analyses were used for the parameters (*z*-average distance from an individual with autism) and (RL-resource load).

### GapMap

By having individual families submit their diagnostic and demographic information through GapMap [18], we can eventually calculate a more accurate prevalence rate with more granularity. A simple Web portal was created to allow individuals with ASD to enter relevant data and to crowdsource from the social and global databases. Caregivers (aged ≥18 years) of persons with a clinical diagnosis of ASD are allowed to submit data, including gender, date of birth, location including city, state, and zip code, specific (clinically defined) diagnosis of an ASD, diagnostic tools, date of diagnosis, any comorbid conditions, email address, and a list of ratable local services used to care for the subject (where local services include medical specialists, therapists, support resources, and “autism-friendly” generic services). We will then validate the disclosed ASD diagnosis by having participants complete a peer-reviewed autism screening measure such as the Social Communication Questionnaire (SCQ) [30] or the Social Responsiveness Scale (SRS) [31]. IP addresses, date and time of submission, and similarity of data submitted will also be used to detect duplicate or flag anomalies as potentially falsified data. These two measures will ensure higher quality data, as crowdsourced-data have been shown to match the quality of expert-curated data with proper instructions for data submission and reasonable validation on input data [32-34].

On validation, we will include the family’s data in GapMap; however, if they do not meet ASD criteria, we will not limit the participant from reviewing local resources. Providing families with an accurate list of local resources gives undiagnosed or untreated individuals more chances for earlier diagnosis and an earlier onset of therapy. In addition, we account for the undiagnosed populations within a community by collecting data from participants who select “No diagnosis, but suspicious” under the clinical ASD diagnosis option.

Mainstream social networking platforms, including Facebook, Twitter, colleague networks, consortia, related events and conferences, websites, and fan-based networks, will be used to reach families who have a family member with a clinical diagnosis of autism.

**Figure 1:**
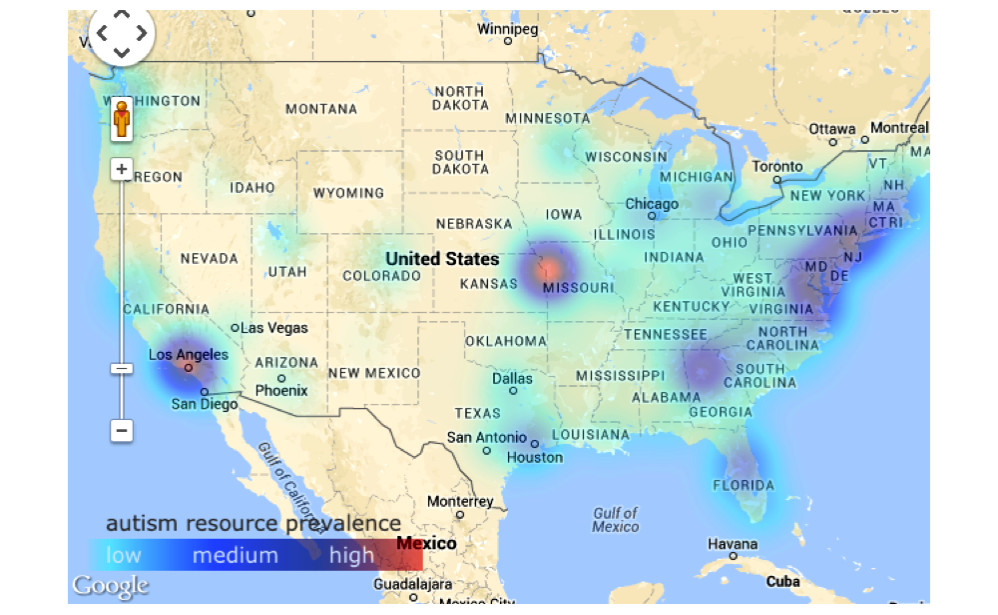
GapMap features an interactive Google heatmap, comparing resource availability to families with a diagnosed individual with autism. The red coloring on the heatmap shows high autism resource prevalence, while purple coloring shows moderate autism resource prevalence, blue coloring shows low autism resource prevalence, and no coloring shows that, based on our calculations, there are very limited autism-resources available.

**Figure 2:**
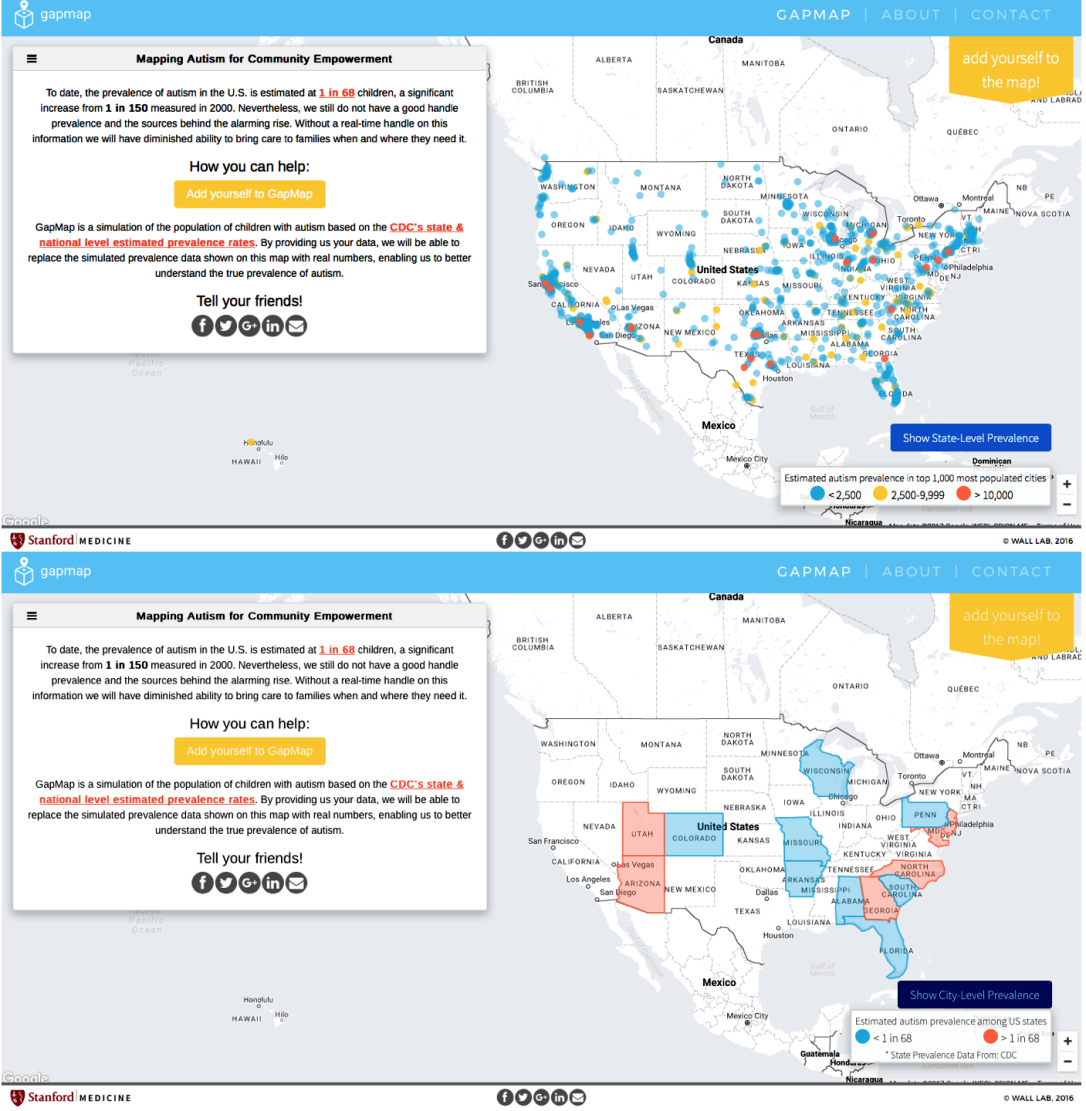
Example of the mapping interface and home page for GapMap (gapmap.stanford.edu). Participants can electronically consent and participate from any mobile device by clicking on the yellow “Add yourself to the map!” button, as well as toggle between country-level and state-level prevalence of diagnosed autism cases.

GapMap’s home page also allows users to search and edit its database of autism resources, as shown in Figure 1. Figure 2, which features a Google map with markers and marker clusters for resources and a heatmap overlay, shows resource availability. The metrics resource load, resource gap, and resource availability were defined to aid in this purpose, and estimates were calculated with limited datasets to provide more evidence for the importance of our tool.

**Figure 3:**
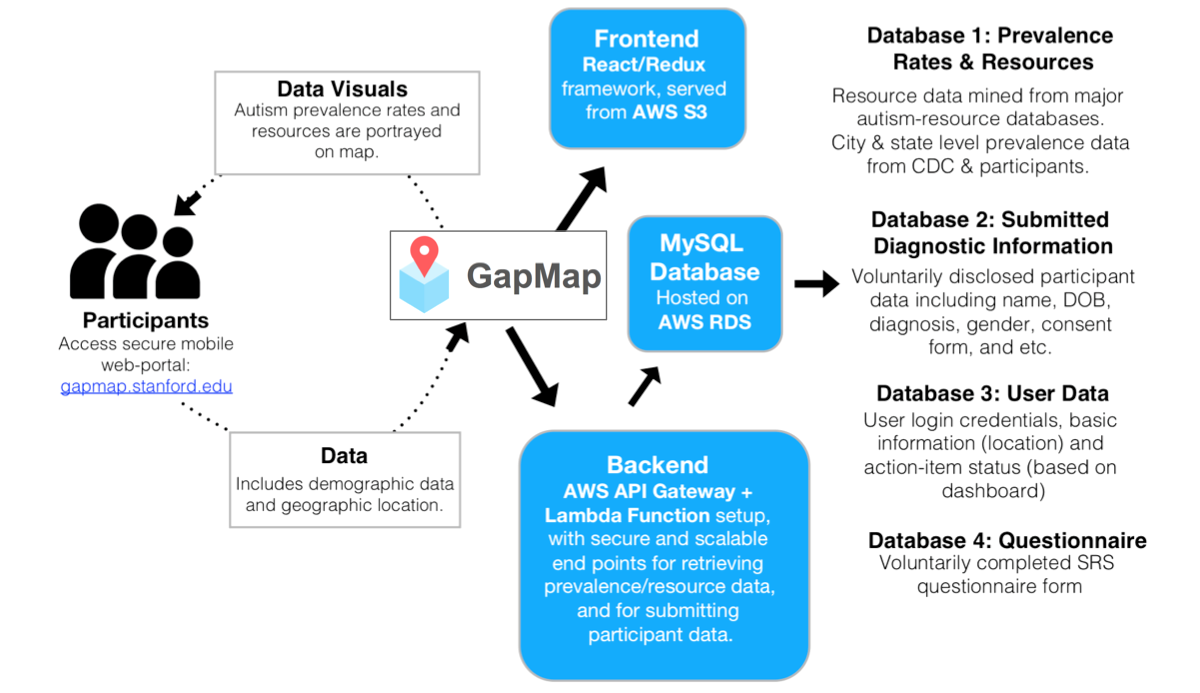
GapMap’s technical architecture. GapMap is hosted on AWS S3, running on a React and Redux front-end framework. The backend framework is comprised of an AWS API Gateway and Lambda Function setup, with secure and scalable end points for retrieving prevalence and resource data, and for submitting participant data. Database 1: unencrypted and stores prevalence rates and resource data; Database 2: encrypted and stores submitted diagnostic information; Database 3: encrypted and stores user login information, location, and action-item status; and Database 4: encrypted and stores the users’ questionnaires.

Data are encrypted and stored on secure MySQL Databases behind a firewall. GapMap is written in React.js and runs on Amazon Web Services Simple Storage Serve (AWS S3). The backend server runs on AWS app program interface (API) Gateway and AWS Lambda. AWS API Gateway executes specific javascript packages, novel code that interacts with our SQL database, on AWS Lambda. The MySQL relational database is hosted on Amazon Relational Database Service (RDS) and consists of four main databases. Database 1 is unencrypted and stores prevalence rates and resource data; Database 2 is encrypted and stores submitted diagnostic information; Database 3 is encrypted and stores user login information, location, and action-item status; and Database 4 is encrypted and stores the users' questionnaires. See Figure 3 for an overview of the system architecture.

## Results

### Average Distance to the Nearest Diagnostic Center

The distance an individual must travel to the nearest autism diagnostic center is important for several reasons. Studies have shown that individuals are less likely to take advantage of medical services when they are located far away, particularly for low-income families [35,36]. Local care is convenient, and families that must travel long distances to diagnostic facilities may wait to observe more risk factors in their child to schedule an appointment [36]. It may also be the case that areas without any autism resources may lack general knowledge of autism or its characteristic behaviors. These delays may dramatically increase the time to diagnosis, as we know to be the case for other disorders in rural regions [37]. However, given the scarcity of autism diagnostic resources, the typical distance individuals travel for an autism diagnosis may be greater than realized.

Our simulation constructed from US Census data revealed the average distance from an individual in the United States to the nearest diagnostic center to be approximately 182 km (50 miles), with a standard deviation of 235 km (146 miles). The list of diagnostic centers included hospitals and clinics with dedicated autism diagnosis personnel, but may have missed many solo and small-town diagnosticians. As complete and precise population maps (at the city level) were not available, the simulation also assumed that populations within counties are uniformly distributed, whereas populations are likely to be clustered even within counties. Both of these limitations may slightly overinflate the resulting distances. Even with a fair amount of overestimation, these distances are alarming, and suggest that autism resource allocation is a central issue that needs to be addressed.

The average distance from an individual with ASD to the nearest diagnostic center was also calculated for the United States and the United Kingdom using locational information scraped from the Internet. The dataset for the United States was comprised of 47,622 individuals with autism and 840 developmental medical centers; the dataset for the United Kingdom was comprised of 737 individuals with autism and 135 diagnostic centers. The pool of individuals sampled was restricted to English speakers with Internet access, which can skew toward cities (where autism resources are likely to be more abundant) and younger adults. The results are shown in Table 1.

**Table 1:** Distance between an individual with autism and the nearest diagnostic center in the United States and United Kingdom.

Location	United States (miles)	United Kingdom (miles)
Average distance	32 km (20)	31 km (19)
Median distance	13 km (8)	11 km (7)
Maximum distance	1,819 km (11,130)	519 km (322)
Percent of individuals living within 30 km (19 miles) of a diagnostic center (*z*)	70%	74%

Notably, the average distances from an individual with autism to the nearest diagnostic center are lesser than the average distance from any individual to the nearest diagnostic center. Although some researchers have hypothesized that this higher rate might be a result of environmental factors in urban areas contributing to this higher incidence [38,39], and distances may be underestimated as a result of biases in sampling methods, these results suggest that individuals who live closer to the diagnostic services are more likely to be diagnosed. This interpretation supports previous research [40], but can also be explained by the incentive for families to move closer to health services if they have concerns for ASD, or other correlational factors such as more advanced parental age (older parents may be more likely to live in urban areas). In order to rule out alternative explanations such as sampling biases or environmental factors and determine whether such an association exists, future work should look at the relationship between age of diagnosis and distance from the nearest diagnostic center. If such a connection does exist, we would expect to see that the average age of diagnosis is lower for individuals closer to a diagnostic provider than for those who must travel extensively. Although we were not able to collect the date of diagnosis for the individuals in this study, the release of GapMap will soon enable such analyses. GapMap will be an effective tool to support or challenge correlations to certain environmental factors or clusters of high autism incidence. As GapMap collects data increasingly, we will also be able to determine the average distance for individual states in the United States.

### Computing Resource Shortages

Although we know autism resources are scarce in many regions, there have not been any attempts to quantify the magnitude or scope of these resource shortages. It will be useful, then, to define several key metrics to determine the existence and magnitude of resource shortages.

**Figure 4:**
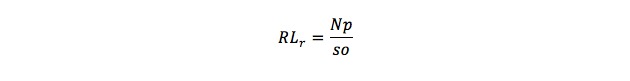
Equation for the resource load for a given resource. Where: r = a given resource; RLr = resource load for a given resource (r); N = the number of individuals nearby; p = the proportion of individuals who are are in need of resources; s = the number of specialists; and o = the number of individuals a specialist can attend yearly.

We will define resource load (RL) as a measure of how well a center can meet the resource demand placed on it. This is a simple ratio of demand to supply, where demand is the number of individuals who require autism services (*Np*) and supply is equal to the product of the number of specialists who work at the center (*s*) and the number of individuals a specialist can attend yearly (*o*). A resource load of 1 would indicate, then, that a center is perfectly able to meet the demand. A resource load of 6, however, means that the center is overburdened with 6 times the number of patients it can comfortably handle. We can expect that centers with a resource load greater than one have longer wait times for appointments. See Figure 4 for the equation.

Resource load may be underestimated for some centers and overestimated for others because individuals are often willing to travel greater distances to receive the “best” medical care for their children. In future, we can use ratings and local services usage information collected from GapMap to help explore the extent of this possible phenomenon by checking for correlations between ratings of and distance traveled to each resource.

**Figure 5:**
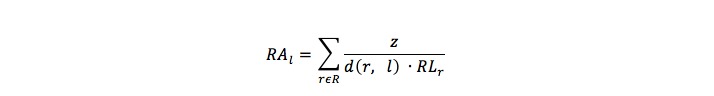
Equation for the resource availability for a given location. Where: l = given location; RAl= resource availability for a given location (l); R = the pool of resource options, where r is one such resource; d(r,l)= the distance between the resource r and the given location (l); RLr = the resource load for the resource r; and z = the average distance an individual is willing to travel for a resource.

Although resource load is useful from the perspective of a resource center, it is also important to consider the availability of resources from the perspective of a family looking for ASD resources. We will therefore define resource availability (RA) with respect to a given location. The equation is shown in Figure 5.

In short, resource availability is the sum of the resources available in a given location, where each resource’s contribution is adjusted by its resource load and its distance to the given location. Resources that are very far away and resources that are overburdened are given little weight. Resource availability will be high when resources with reasonable resource load are nearby, and will be at least equal to one if one resource exists within a reasonable distance with a resource load of one. A resource gap, or region in which there exist limited diagnostic or treatment resources with respect to the demand, is indicated by a resource availability of less than 1. These regions are where we should focus most on improving the accessibility of resources.

The parameter *z* accounts for how far the average individual will travel to seek out a resource. Note that this distance may vary with the type of resource—for example, individuals may be willing to travel farther for behavioral therapy (once a child has been diagnosed) than for diagnostic resources, when they are acting only on suspicions of developmental delays. For this study, we estimate *z* for diagnostic centers at 30 km by using the results of our previous analysis, where we estimate that 70% of individuals with autism in the United States live within 30 km of a diagnostic center.

We computed resource load for 840 developmental medical centers in the United States. About 0.5% of the centers had a resource load of less than one, and even after giving some leeway to account for the limited information on diagnosticians and diagnostic centers, only 5% of the centers had a resource load of less than 5. Alarmingly, 18% of centers had a resource load greater than 25, or experienced 25 times the demand they could handle. The average center had a resource load of 18 and a standard deviation of 14. The median center had a resource load of 14, and the maximum resource load experienced by a center was 113. These numbers are distressing, but not particularly surprising, given the difficulty and time involved in obtaining an autism diagnosis. Regardless, they make a convincing case for further research in and reassessment of our investment in diagnostic resources.

## Discussion

### Principal Findings

We are missing the information about diagnostic capabilities in smaller facilities and rural areas. Specialists ranging from general practitioners to developmental pediatricians can (but are not required to) be certified to diagnose ASD, and most use brief, unstandardized assessment instruments to diagnose ASD [15,26]. Not all certified specialists feel comfortable giving a diagnosis and will refer parents to another professional for diagnosis [26]. Because of this, it is difficult to know which clinicians are also autism diagnosticians. This phenomenon is particularly true in rural areas, where facilities dedicated specifically to ASD are few or nonexistent and generalist clinicians are more prevalent [13,14]. This is problematic because parents who are referred from their pediatrician to a specialist are more likely to receive a diagnosis sooner [14]. In these very rural areas, parents may need to rely on a family doctor or pediatrician to recognize risk factors or early signs of ASD. Soon, we will be able to use GapMap to learn more about these complex dynamics and come to a better understanding of autism diagnoses, small town diagnosticians, and rural specialists who provide referrals.

Although these analyses and metrics highlight the lack of resources in much of the United States and the overburdening of many centers, they are not enough. We have built GapMap as a tool to collect important information and visually display the results. The collected location information, diagnosis, diagnostic tools, and comorbid conditions will be used to obtain both widespread and highly localizable autism prevalence rates. Date of diagnosis and age will be aggregated and used to obtain localizable average age of diagnosis, a measure that correlates with difficulty obtaining a diagnosis and can be used to help approximate geographic differences in resource accessibility. We will also collect the specific diagnoses of individuals who submit their data, including diagnostic and statistical manual of mental disorders (DSM-IV and DSM-5) diagnoses and an option for “no diagnosis, but suspicious.” This information will allow us to track individuals who remain undiagnosed, providing us with valuable data that is often overlooked in studies of prevalence rates. Ratings and local services will be used to estimate resource usage trends with respect to geography and resource density. Prevalence rates and local service usage will also be used to calculate resource load and availability for different resource types such as behavioral therapy.

### Conclusions

There is a dearth of ASD resources, as well as a lack of understanding of the extent and epidemiology of these resource gaps. Statistics computed from simulations and web scraping suggest that individuals located close to diagnostic centers are more likely to be diagnosed. By quantitatively defining resource load and resource availability, we provide a means to measure and enable the alleviation of increasingly overburdened diagnostic centers and resource-poor areas where parents are unable to diagnose their children as quickly and easily as needed. The release of GapMap will collect crucial information that will provide more accurate data for computing resource loads and availability, uncovering the impact of resource epidemiology on age and likelihood of diagnosis, and gathering localized autism prevalence rates, both from families that have already received a diagnosis and families that haven’t received an official diagnosis yet.

## Abbreviations

API: application program interface
ASD: Autism spectrum disorders
AWS S3: Amazon Web Services Simple Storage Serve
CDC: The Centers for Disease Control and Prevention
DSM: diagnostic and statistical manual of mental disorders
RDS: Amazon Relational Database Service
SCQ: Social Communication Questionnaire
SRS: Social Responsiveness Scale
